# Adipocytokines, C-Reactive Protein, and Cardiovascular Disease: A Population-Based Prospective Study

**DOI:** 10.1371/journal.pone.0128987

**Published:** 2015-06-02

**Authors:** Ekim Seven, Lise L. N. Husemoen, Thomas S. G. Sehested, Hans Ibsen, Kristian Wachtell, Allan Linneberg, Jørgen L. Jeppesen

**Affiliations:** 1 Department of Internal Medicine, Glostrup Hospital, University of Copenhagen, Glostrup, Denmark; 2 Research Centre for Prevention and Health, the Capital Region of Denmark, Glostrup, Denmark; 3 Department of Cardiology P, Gentofte Hospital, University of Copenhagen, Gentofte, Denmark; 4 Department of Internal Medicine, Holbæk Hospital, University of Copenhagen, Holbæk, Denmark; 5 Faculty of Health and Medical Sciences, University of Copenhagen, Copenhagen, Denmark; 6 Department of Clinical Experimental Research, Glostrup Hospital, Glostrup, Denmark; Virginia Commonwealth University, UNITED STATES

## Abstract

**Background:**

Being overweight or obese is associated with a greater risk of coronary heart disease and stroke compared with normal weight. The role of the specific adipose tissue-derived substances, called adipocytokines, in overweight- and obesity-related cardiovascular disease (CVD) is still unclear.

**Objective:**

To investigate the associations of three adipose tissue-derived substances: adiponectin, leptin, and interleukin-6 with incident CVD in a longitudinal population-based study, including extensive adjustments for traditional and metabolic risk factors closely associated with overweight and obesity. C-reactive protein (CRP) was used as a proxy for interleukin-6.

**Methods:**

Prospective population-based study of 6.502 participants, 51.9% women, aged 30–60 years, free of CVD at baseline, with a mean follow-up time of 11.4 years, equivalent to 74,123 person-years of follow-up. As outcome, we defined a composite outcome comprising of the first event of fatal and nonfatal coronary heart disease and fatal and nonfatal stroke.

**Results:**

During the follow-up period, 453 composite CV outcomes occurred among participants with complete datasets. In models, including gender, age, smoking status, systolic blood pressure, treatment for hypertension, diabetes, body mass index (BMI), total cholesterol, high-density-lipoprotein cholesterol, homeostasis model assessment of insulin resistance, estimated glomerular filtration rate, adiponectin, leptin, and CRP, neither adiponectin (hazard ratio [HR] with 95% confidence interval [CI]: 0.97 [0.87–1.08] per SD increase, *P* = 0.60) nor leptin (0.97 [0.85–1.12] per SD increase, *P* = 0.70) predicted the composite outcome, whereas CRP was significantly associated with the composite outcome (1.19 [1.07–1.35] per SD increase, *P* = 0.002). Furthermore, in mediation analysis, adjusted for age and sex, CRP decreased the BMI-associated CV risk by 43% (95%CI 29–72).

**Conclusions:**

In this study, neither adiponectin nor leptin were independently associated with CVD, raising questions over their role in CVD. The finding that CRP was significantly associated with an increased risk of CVD and decreased the BMI-associated CVD risk substantially, could imply that interleukin-6-related pathways may play a role in mediating overweight- and obesity-related CVD.

## Introduction

Being overweight (body mass index [BMI] ≥25.0 to 29.9 kg/m^2^) is associated with a greater risk of coronary heart disease (CHD) and stroke compared with normal weight, and being obese (BMI ≥30.0 kg/m^2^) is associated with a greater risk of CHD and stroke compared with overweight [1]. It is a matter of great concern that these associations point towards a potentially ever-growing public health threat due to the escalating global epidemic of overweight and obesity [2].

It is well-known that people who are overweight or obese tend to have an adverse cardiovascular disease (CVD) risk profile with raised blood pressure, cholesterol, and glucose, and a substantial part of the excess CVD risk of being overweight or obese are mediated by these three risk factors [1].

During the last decades, it has become evident that it is the excess of adipose tissue, and not muscle tissue, that confers an increased health risk to the overweight and the obese, and that adipose tissue is not just a passive energy-conserving tissue [3–7]. On the contrary, adipose tissue is a very active tissue that secretes various pro-inflammatory, vasoactive, and metabolic active hormones and cytokines, which collectively have been called adipocytokines or just adipokines [3–7]. Based on both animal and human studies, it has been proposed that these adipose tissue-derived substances could play a pivotal role in overweight-and obesity-related diseases, such as atherosclerosis, hypertension, and type 2 diabetes, which could eventually lead to overt CVD [3–7]. Among the various adipocytokines, there has been considerable emphasis on leptin, adiponectin, and interleukin-6 [3–7] that stimulates production of C-reactive protein (CRP) by the liver [8]. Nevertheless, the results linking adipose tissue-derived factors to overt CVD in humans have been conflicting [4–7], and there is still considerable uncertainty as to whether these substances can explain a portion of the residual CVD risk related to being overweight or obese [4–7].

Therefore, based on the aforementioned issues, we initiated this study hoping to shed some new light on the putative role of adipocytokines in human CVD, having both CVD pathophysiology and CVD risk markers (on top of an established CVD risk score) in mind. More specifically, we studied the associations of adiponectin, leptin, and the interleukin-6 product CRP (used as a proxy for interleukin-6) with incident CHD and stroke in the Inter99 study [9]. This prospective population-based study is particularly well-suited to investigate the actual impact of adipocytokines on CVD risk, because the Inter99 study, in addition to the traditional CVD risk factors, includes several important potential confounders and mediators of overweight- and obesity-related CVD diseases, such as serum insulin, estimates of insulin resistance, a stable measurement of long-term glucose control (HgA1c), and estimated glomerular filtration rate (both adiponectin and leptin are partially biodegraded and/or eliminated by the kidney) [10–14].^.^

## Methods

### Study Population

The current study utilized data from the Inter99 study [9,15]. In brief, Inter99 is a population-based randomized, non-pharmacological intervention study (CT00289237, ClinicalTrials.gov), designed to investigate the effects of lifestyle interventions for prevention of CVD. The intervention group consisted of 13,016 individuals aged 30–60 years, recruited from the Danish Central Personal Register as an age- and sex-stratified random sample of the population living in 11 municipalities in the former Copenhagen County. A total of 6,784 (52.5%) persons attended the baseline examination, which included a validated self-administered questionnaire, physical examination, and blood tests. Participants were randomized into two groups (A and B) and were all given a lifestyle consultation with personal health advice. Furthermore, participants from group A with high risk of ischemic heart disease (IHD) were offered group-based lifestyle counselling on smoking cessation, increased physical activity, and healthier dietary habits. Details on the study and intervention program, including information on factors determining response to participation, have been described elsewhere [9,15,16]. The present study population included 6,502 persons, free of prior CVD, with measurements of either adiponectin, leptin, or CRP. Data were considered observational and analyses were adjusted for study group. The Inter99 study was approved by the local (Copenhagen County) ethical committee (KA 98 155) and was conducted in accordance with the Declaration of Helsinki. All participants gave a written informed consent before taking part of the study.

### Main Outcome of Interest: CVD

All residents in Denmark have a unique and permanent personal civil registration number, which allows linkage at an individual level of data from national registers. Information about CVD death was obtained from the Danish Registry of Causes of Death [17]. Information on diseases was obtained from the Danish National Patient Register [18], which has information on all admissions to Danish hospital since 1978. The included diagnoses were the *International Classification of Diseases* (ICD) ICD10 and ICD8 diagnoses involving IHD (ICD10: I20–25 and ICD8:410–414) and stroke (ICD10: I60–69 and ICD8: 431, 433–434. and 436). We defined three outcomes: a diagnosis of or death caused by IHD, a diagnosis of or death caused by stroke and a composite outcome, which included all the above-mentioned diagnoses.

### Exposure Measurements

The invitation included a validated questionnaire to be completed in advance wherein data about lifestyle, education, working conditions, chronic diseases, use of medication, among others were recorded [9,15,16,19]. Alcohol intake was registered as numbers of standard drinks (12 g alcohol) per week. Leisure time physical activity was categorized as sedentary, moderate activity, regular exercise, and regular hard exercise (the last two categories were merged to high). Based on their dietary habits participants were classified into three groups: healthy, normal, and unhealthy. Smoking habits was recorded as never, former, or current. Height was measured without shoes to the nearest centimeter, weight without shoes and overcoat to the nearest 0.1 kg. BMI was calculated as weight in kilograms divided by height in meters squared. Waist circumference was measured midway between the lower rib margin and the iliac crest to the nearest centimeter [9]. Heart rate (beats/minute) was derived from the electrocardiogram. All participants had their blood pressure measured twice, using a mercury sphygmomanometer (Mercuro 300; Speidel & Keller GmpH & Co, Jungingen, Germany) with appropriate cuff size, after 5 min of rest, in the lying position. If systolic blood pressure was at least 140 mmHg or diastolic blood pressure at least 90 mmHg, the measurements were repeated twice to minimize the ‘white-coat’ effect with the two lowest values being recorded, and the average of the recorded measurements was used.

### Blood Assays

Fasting blood samples were drawn and on a daily basis transferred to the laboratory at Steno Diabetes Center, Gentofte, Denmark. Total cholesterol, high-density lipoprotein (HDL) cholesterol, triglycerides, and plasma glucose were determined by enzymatic techniques (Boehringer Mannheim, Germany). HgA1c was measured using the high-performance liquid chromatography method [9]. Serum insulin was measured with flourimmunoassay technique [9]. Adiponectin, leptin, and CRP were analyzed at Tethys Bioscience (Emeryville, California, USA) using an ultrasensitive molecular counting technology platform (Singulex, St Louis, Missouri, USA) [20]. Reagents were obtained from R&D Systems (Minneapolis, Minnesota, USA) and U.S. Biological (Swampscott, Massachusetts, USA). Biomarker concentrations were calculated as the mean of 3 replicates. Assays had dynamic range of 10^2^–10^3^, intraplate coefficients of variation of 5% or and an average lower limit of detection of 10 pg/mL [20].

### Statistics

We performed all analyses with SAS version 9.3 (SAS Institute, Cary, North Carolina, USA). Data are presented as mean ± SD for normally distributed variables, as the median (5^th^ to 95^th^ percentile) for skewed distributed variables, and frequency in percentage for categorical variables. Group comparisons were done with the ANOVA test for normally distributed variables, the chi-square test for categorical variables, and the Kruskal-Wallis test for skewed distributed variables (Table 1). We performed multivariate Cox regression analyses to determine the associations between baseline circulating concentrations of adiponectin, leptin, and CRP with CVD. In the various models, missing data were handled with the pairwise deletion technique, which involves deleting a case when the case is missing a variable required for a particular analysis, but including the case in analyses for which all required variables are present. Therefore, number of participants varies among models. In model 1, we adjusted for sex, age, and intervention group. In model 2, we added adjustments for the Framingham Risk CVD Score variables (total cholesterol, HDL cholesterol, smoking status, systolic blood pressure, treatment of hypertension, and baseline diabetes) [21]. Model 2 was designed to evaluate the usefulness of adiponectin, leptin, and CRP as CVD risk markers on top of an established risk score program used in clinical practice. In model 3, we further adjusted for BMI, homeostasis model of insulin resistance (HOMA-IR) [22], and estimated glomerular filtration rate (eGFR) [23]. We present hazard ratios (HR) with 95% confidence intervals (CI) per SD increase in log transformed serum adiponectin, leptin, and CRP concentrations. The log-rank test was used to compare event-free survival across baseline concentrations of adiponectin, leptin, and CRP divided into sex-specific tertiles. We used age as underlying time axis and delayed entry where participants enter the analysis at their baseline age, and they exited the analysis at their event or censoring age. If participants experienced multiple events, only the first event was included. The linearity assumption was checked by adding the term squared and cubed to the model and checking for significance. The proportional hazards assumption was checked visually. The predictive performance of model 2, the Framingham Risk Score model, with and without variables of interest, were summarized using C statistics estimated from logistic regression models and further assessed with Net Reclassification Improvement (NRI) and Integrated Discrimination Improvement (IDI), by means of a SAS macro as proposed by Pencina et al. [24], using risk cut points of 5% and 15%. Furthermore, we estimated the percentage of excess risk mediated (PERM) for the different risk factors and mediators with the formula: *Perm = ((HR confounder adjusted—HR confounder and mediator adjusted) / (HR confounder adjusted -1)) x 100* [25]. Interactions between variables of interest and sex, age, and BMI were examined, and none was found. All statistical tests were two-sided and the significance level was *P<0*.*05*.

**Table 1:** Baseline Characteristics of Participants in Inter99.

Variables	All *(n = 6*,*502)*	Cases *(n = 527)*	Controls *(n = 5*,*975)*
**Demographic**			
Female sex, %	51.9	42.3	52.7***
Age, years	45.9 ±7.9	49.1 ±7.3	45.6 ±7.9***
Self-reported diabetes, %	1.9	5.4	1.6***
High educational level, %	28.8	25.1	29.1*
Use of antihypertensive drugs, %	6.0	14.0	5.2***
Use of cholesterol lowering drugs, %	3.0	3.7	2.9
**Life style**			
Alcohol intake, standard drinks/week	6 (0–32)	7 (0–39)	6 (0–32)*
High physical activity, %	40.4	38.5	40.6
Unhealthy dietary habits, %	15.6	21.1	15.1***
Smoking, %			
Never	35.4	25.7	36.3***
Former	25.6	27.8	25.4
Current	39.0	46.5	38.3***
**Anthropometric**			
Body mass index, kg/m^2^	26.3 ±4.6	27.3 ±4.9	26.2 ±4.6***
Waist circumference, cm	86 ±13	90 ±14	86 ±13***
**Hemodynamic**			
Heart rate, beats/min	67 ±11	68 ±11	67 ±11*
Systolic blood pressure, mm Hg	130 ±17	137 ±20	129 ±17***
Diastolic blood pressure, mm Hg	82 ±11	86 ±13	82 ±11***
**Lipid**			
Total cholesterol, mmol/L	5.5 ±1.1	6.0 ±1.3	5.5 ±1.1***
HDL-Cholesterol, mmol/L	1.43 ±0.4	1.36 ±0.4	1.44 ±0.4***
Triglycerides, mmol/L	1.1 (0.5–2.9)	1.3 (0.6–3.8)	1.0 (0.5–2.8) ***
**Metabolic and renal**			
CRP, mg/L	0.9 (0.1–7.6)	1.2 (0.2–9.9)	0.9 (0.1–7.3) ***
Leptin all, ng/mL	5.7 (0.9–33.1)	5.6 (1.1–31.5)	5.7 (0.9–33.1)
Leptin men, ng/mL	3.2 (1–13)	3.9 (1–16)	3.1 (1–13)**
Leptin female, ng/mL	10.4 (2–44)	11.5 (3–44)	10.2 (2–43)
Adiponectin, μg/mL	6.9 (2–26)	6.3 (2–25)	7.0 (2–26)*
HOMA-IR, units	1.4 (0.5–4.4)	1.6 (0.6–6.1)	1.3 (0.5–4.4) ***
HbA1c, %	5.9 ±0.6	6.1 ±0.9	5.8 ±0.6***
FPG, mmol/l	5.6 ±1.1	6.0 ±1.6	5.6 ±1.1***
Estimated GFR, ml/min/1.73m^2^	88 ±19	88 ±21	88 ±19

Data are presented as mean ±standard deviation for normally distributed variables, as median (5^th^–95^th^ percentile) for skewed distributed variables, and frequency in percent for categorical variables. CVD indicates cardiovascular disease; HDL, high-density lipoprotein; CRP, C-reactive protein; HOMA, homeostatic model assessment; IR, insulin resistance; HbA1c, hemoglobin A1c; FPG, fasting plasma glucose; GFR, glomerular filtration.

*P <0.*05*

**P <0.*01*

***P <0.001. *P* is for difference between CVD cases and controls.

## Results

Table 1 summarizes baseline characteristics of the study population according to the occurrence of the composite outcome during the follow-up period. The 6,502 participants had a mean follow-up time of 11.4 years, equivalent to 74,123 person-years of follow-up. We registered 346 incident cases of IHD (including 118 cases with acute myocardial infarction, the remaining being primarily stable and unstable angina pectoris), 208 incident cases of stroke, and 238 deaths caused by IHD or stroke. Participants, who developed a first CVD event, had a higher baseline prevalence of the traditional CVD risk factors compared with participants without events. With respect to adipocytokines, participants with composite outcomes had higher baseline CRP and lower adiponectin concentrations compared with event-free participants. Because women, on any given measure of obesity, have approximately 2.5-fold higher leptin concentrations compared with men [26], we performed a sex-stratified analysis that showed that men with events had higher baseline leptin concentrations compared with men without events, whereas this was not the case for women.

Table 2 shows HRs with 95%CI for the associations of adiponectin with the various CVD events adjusted models. In univariate model, we found a HR of 0.89 ([95%CI 0.82–0.97], *P = 0*.*010*) per one SD increase in baseline adiponectin concentrations for the combined events, but as seen in Table 2, we found only one significant association in the adjusted models. Accordingly, adiponectin was associated with incident IHD with a HR of 0.89 ([95%CI 0.80–0.99], *P = 0*.*039*) in model 1.

**Table 2:** Associations of Adiponectin with Incident Cardiovascular Disease in Inter99.

	Model 1 *(n = 5*,*950)*		Model 2 *(n = 5*,*895)*		Model 3 *(n = 5*,*763)*	
	Events	HR (95% CI)	Events	HR (95% CI)	Events	HR (95% CI)
**Combined events**	470	0.94 (0.86–1.03)	463	1.00 (0.92–1.11)	453	0.97 (0.87–1.08)
**IHD**	309	0.89 (0.80–0.99)*	305	0.97 (0.86–1.09)	297	0.95 (0.83–1.09)
**Stroke**	182	1.01 (0.87–1.16)	181	1.03 (0.89–1.21)	179	0.95 (0.80–1.13)

Data are presented as Hazard Ratio per one standard deviation increase in log transformed adiponectin level. **Model 1:** Adjusted for sex, age and intervention group. **Model 2:** Model 1 + total cholesterol, HDL-cholesterol, smoking status, systolic blood pressure, treatment for hypertension and baseline diabetes. **Model 3:** Model 2 + BMI, HOMA-IR, eGFR, CRP and leptin.

*P <0.*05*

Table 3 shows HRs with 95%CI for the associations of leptin with the various CVD events in adjusted models. In univariate analysis, we found a HR of 1.02 ([95%CI 0.94–1.10], *P = 0*.*64*) per one SD increase in baseline leptin concentrations for the combined event, and even though some associations researched statistical significance in model 1, the associations lost statistical significance in model 2 and 3.

**Table 3:** Associations of Leptin with Incident Cardiovascular Disease in Inter99.

	Model 1 *(n = 5*,*854)*		Model 2 *(n = 5*,*800)*		Model 3 *(n = 5*,*763)*	
	Events	HR (95% CI)	Events	HR (95% CI)	Events	HR (95% CI)
**Combined events**	464	1.17 (1.07–1.28)*	457	1.02 (0.93–1.13)	453	0.97 (0.85–1.12)
**IHD**	303	1.18 (1.05–1.32)*	299	0.98 (0.87–1.11)	297	0.95 (0.80–1.12)
**Stroke**	182	1.15 (0.99–1.33)	181	1.10 (0.94–1.29)	179	1.08 (0.87–1.35)

Data are presented as Hazard Ratio per one standard deviation increase in log transformed leptin level. **Model 1:** Adjusted for sex, age and intervention group. **Model 2:** Model 1 + total cholesterol, HDL-cholesterol, smoking status, systolic blood pressure, treatment for hypertension and baseline diabetes. **Model 3:** Model 2 + BMI, HOMA-IR, eGFR, CRP and adiponectin.

*P <0.*01*

Because it could be hypothesized that the leptin-to-adiponectin ratio could serve as a CVD risk index superior to leptin or adiponectin alone, we also studied the associations of this ratio with the various CVD events, as depicted in Table 4. However, no statistical significant associations were found between the leptin-to-adiponectin ratio and CVD in model 2 and 3. Furthermore, normalization of adiponectin and leptin to BMI did not produce significant results either in model 2 and 3 (*P>0*.*55*) (data not shown*)*.

**Table 4:** Associations of Leptin/Adiponectin Ratio with Incident Cardiovascular Disease in Inter99.

	Model 1 *(n = 5*,*854)*		Model 2 *(n = 5*,*800)*		Model 3 *(n = 5*,*763)*	
	Events	HR (95% CI)	Events	HR (95% CI)	Events	HR (95% CI)
**Combined events**	464	1.20 (1.09–1.32)*	457	1.02 (0.91–1.13)	453	1.00 (0.88–1.15)
**IHD**	303	1.26 (1.13–1.42)*	299	1.00 (0.89–1.15)	297	1.00 (0.86–1.19)
**Stroke**	184	1.12 (0.97–1.30)	181	1.07 (0.90–1.26)	179	1.08 (0.88–1.33)

Data are presented as Hazard Ratio per one standard deviation increase in log transformed Leptin/Adiponectin ratio. **Model 1:** Adjusted for sex, age and intervention group. **Model 2:** Model 1 + total cholesterol, HDL-cholesterol, smoking status, systolic blood pressure, treatment for hypertension and baseline diabetes. **Model 3:** Model 2 + BMI, HOMA-IR, eGFR and CRP.

*P <0.*001*

**Fig 1:**
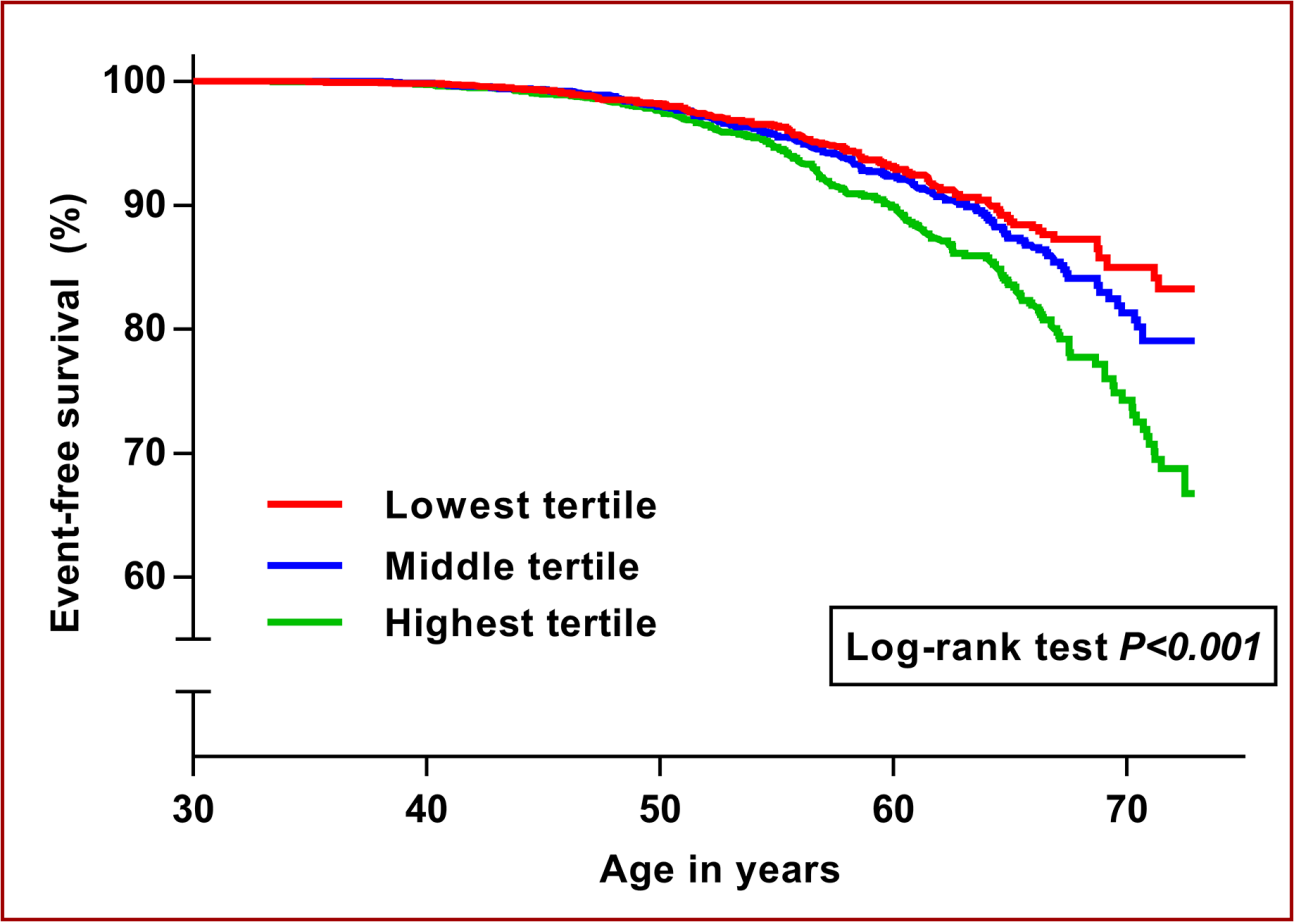
C-reactive protein and event-free survival. The figure shows Kaplan-Meier curves for event-free survival stratified by sex-specific tertiles of baseline C-reactive protein in Inter99.

Table 5 displays HRs with 95%CI for the associations of CRP with the various CVD events in adjusted models. In univariate model, we found a HR of 1.32 ([95%CI 1.21–1.44], *P<0*.*001*) per one SD increase in baseline CRP concentrations for the combined event, and the HRs in model 1 for all 3 outcomes were basically similar to the aforementioned univariate HR. Furthermore, although the strength of the associations between CRP and the 3 events were attenuated in model 2 and 3, they were still statistically significant. Furthermore, with respect to CRP and CVD, Fig 1 displays Kaplan-Meier plot for baseline CRP concentrations in sex-specific tertiles plotted against event-free survival time. Cut points for CRP tertiles for men were less than 0.49 mg/L (median = 0.27), between 0.49 mg/L and 1.37 mg/L (median = 0.82) and above 1.37 mg/L (median = 2.64). For women the corresponding figures were less than 0.55 mg/L (median = 0.28), between 0.55 mg/L and 1.68 mg/L (median = 0.96) and above 1.68 mg/L (median = 3.53). The curves start to separate before the age of 60 with those participants in the highest CRP tertile having the worst outcome.

**Table 5:** Associations of CRP with Incident Cardiovascular Disease in Inter99.

	Model 1 *(n = 6*,*193)*		Model 2 *(n = 6*,*138)*		Model 3 *(n = 5*,*763)*	
	Events	HR (95% CI)	Events	HR (95% CI)	Events	HR (95% CI)
**Combined events**	500	1.33 (1.22–1.45)***	493	1.18 (1.08–1.30)***	453	1.19 (1.07–1.33)**
**IHD**	331	1.32 (1.19–1.47)***	327	1.14 (1.02–1.27)*	297	1.15 (1.00–1.32)*
**Stroke**	194	1.32 (1.15–1.52)***	191	1.23 (1.06–1.42)**	179	1.08 (0.88–1.33)

Data are presented as Hazard Ratio per one standard deviation increase in log transformed CRP level. **Model 1:** Adjusted for sex, age and intervention group. **Model 2:** Model 1 + total cholesterol, HDL-cholesterol, smoking status, systolic blood pressure, treatment for hypertension and baseline diabetes. **Model 3:** Model 2 + BMI, HOMA-IR, eGFR, adiponectin and leptin.

*P <0.*05*

**P <0.*01*

***P <0.001

If we added CRP to model 2, C statistics increased from 0.697 to 0.701 though the change was not significant (*P = 0*.*26*). However, we saw a small, but statistically significant, improvement in reclassification with NRI of 0.039 (*P = 0*.*012*) and IDI of 0.003 (*P<0*.*001*).

Finally, no effect modification by sex was observed for the associations of adiponectin, leptin, and CRP (*P* for interaction = *0*.*10*, *0*.*32*, *and 0*.*99*, respectively) with incident CVD in any of the fully adjusted models.

### Supplemental Analyses

Since it is presence of low-grade chronic inflammation that has been hypothesized to be involved in the pathophysiology of CVD [8], we excluded 182 participants with CRP more than 10 mg/L (indicative of ongoing subclinical or clinical infectious or inflammatory illness) without observing any change in the associations. Additionally, because smoking causes a low-grade inflammatory state, associated with higher CRP (in our study: median 1.1 mg/L vs. 0.8 mg/L, *P<0*.*001*) and lower BMI (in our study: mean 25.1 kg/m^2^ vs 25.7 kg/m^2^, *P<0*.*001*) [8], we repeated the analyses excluding all smokers. Among 3781 non-smokers, 266 combined CVD events occurred, and in the non-smoking population CRP was still associated with CVD in the fully adjusted model 3 (HR with 95%CI: 1.18 [1.02–1.37], P = 0.025). In this context, it is worthy of note that higher BMI was also (as expected) associated with higher CRP [8] in our study population (median [5–95% percentile] for BMI less than 25.0 kg/m^2^, BMI at least 25.0 and less than 30.0 kg/m^2^, and BMI equals to or more than 30.0 kg/m^2^: 0.6 mg/L [0.1–5.3], 0.9 mg/L [0.2–6.9], and 2.2 mg/L [0.4–14.2], *P<0*.*001*, respectively).

Because cholesterol-lowering drugs could have anti-inflammatory effects [8], we repeated all the analyses after excluding 60 participants, who were on statin treatment, and again the results were unchanged.

In model 3, when we replaced HOMA-IR with HbA1c, as a measure of the average plasma glucose concentration over a prolonged period, we obtained similar results.

**Fig 2:**
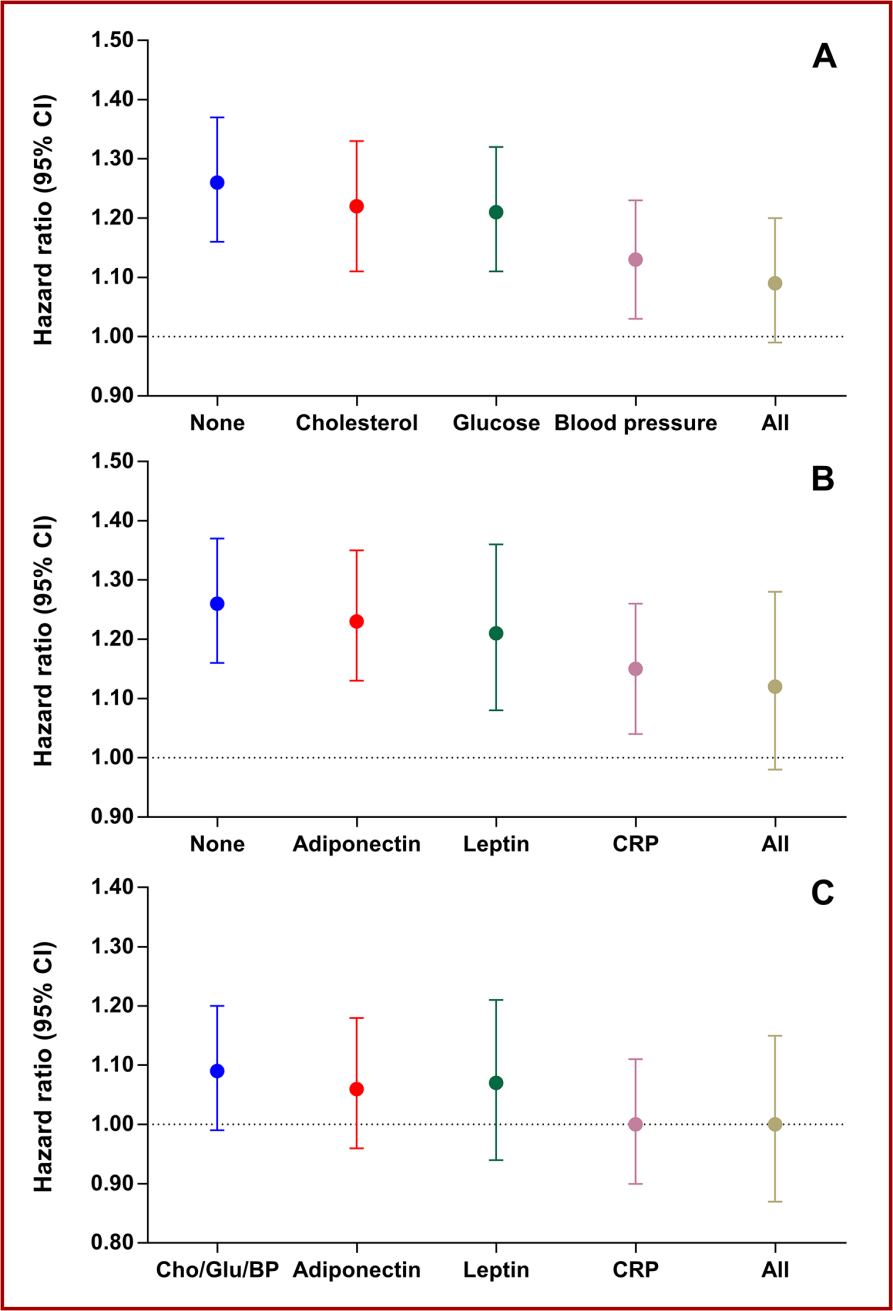
Mediators of body mass index-related cardiovascular disease risk. The figure shows hazard ratios per 5 kg/m^2^ higher body mass index adjusted for sex and age and different combinations of mediators for the combined event. Panel A shows the mediating effects of glucose, cholesterol, and blood pressure, and their combination. Panel B shows the mediating effects of adiponectin, leptin, and C-reactive protein (CRP), and their combination. Panel C shows the mediating of adiponectin, leptin, and C-reactive protein, and their combination on the residual body mass index-related risk already adjusted for sex, age, glucose (Glu), cholesterol (Chol), and blood pressure (BP).

Finally, because the focus of this paper was metabolic mediators of the effects of overweight and obesity on CHD and stroke, we calculated HRs with 95%CI per 5 kg/m^2^ higher BMI adjusted for sex and age and with further adjustments for different combinations of possible mediators in CHD and stroke. The results of these calculations are shown in Fig 2. Among the traditional overweight- and obesity-related CVD risk factors (Fig 2A), systolic blood pressure decreased BMI-associated risk the most (PERM = 51% [95%CI 36–81]), and with addition of cholesterol and fasting glucose to systolic blood pressure the BMI-associated risk decreased further (PERM = 66% [95%CI 47–100]). Among the adipose tissue-derived substances (Fig 2B), adiponectin (PERM = 9% [95%CI 0–20]) and leptin (PERM = 19% [95%CI 2–52]) had some effect on the BMI-associated risk, whereas CRP decreased the BMI-associated CVD risk substantially (PERM = 43% [95%CI 29–72]). Additionally, in a final model (Fig 2C) including sex, age, fasting glucose, total cholesterol, systolic blood pressure, and further separate adjustments for adiponectin, leptin, and CRP, addition of CRP eliminated the BMI-associated CVD risk (PERM = 100% [95%CI 45–100]), whereas adiponectin and leptin had basically no effect.

## Discussion

The principal findings of this study are that neither adiponectin nor leptin were useful markers for increased CVD risk in Inter99, raising questions over their role in CVD. In contrast, the interleukin-6 product CRP may be a useful marker for increased CVD risk, and conditions leading to higher CRP concentrations (in the low-grade inflammation range) may play a role in the pathophysiology of CVD. In addition, the present study suggests, as seen in Fig 2C, that a substantial part of the increased CVD risk associated with overweight and obesity may be attributed to four factors: higher blood pressure, glucose, cholesterol, and interleukin-6-related pathways.

A causal role of adiponectin in CVD is controversial [4–7]. Numerous animal studies have reported that adiponectin has anti-inflammatory, antiatherogenic, antihypertensive, and anti-diabetic/insulin-sensitizing properties [3–7,27,28], and because overweight and obese persons have lower circulating adiponectin concentrations compared with normal weight persons [3–7], it has been proposed that a low amount of adiponectin may play a significant role in overweight- and obesity-related CVD [3–7]. However, numerous epidemiological studies, as summarized in three recently published independent meta-analyses [29–31], have not found any real evidence to suggest that lower circulating adiponectin concentrations are related to an increased risk for CHD or stroke. In this perspective, our results are in agreement with the medical literature.

A casual role of leptin in CHD and stroke is not clear [32], although animal studies in particular have related adverse CV properties to leptin [3–7]. Data relating leptin to CVD has been inconsistent, and the positive association reported have been largely dependent on BMI [32]. Nevertheless, a recently published systematic review and meta-analysis, comprising of eight original articles with a total of 21,064 participants and 2,053 CHD events [33] reported a positive association between leptin and incident CHD after adjustment for age, sex, lipids, blood pressure, and BMI, although the association did not quite reach statistical significance [33]. In contrast, a recent update from the Jackson Heart Study, which was not included in the systematic review and meta-analysis [33], reported that, among 4,571 Jackson Heart Study participants, followed for an average of 6.2 years, with occurrence of 98 incident CHD and 87 incident ischemic stroke events, leptin was not associated with incident CHD or incident stroke [34], So, at first glance based on the available evidence, including our data, leptin does not seem to be an independent predictor CVD. Nonetheless, in the context of leptin and CVD, the possibility of over-adjustment bias needs to be briefly discussed [35]. Over-adjustment bias is defined as control for an intermediate variable on a causal path from exposure to outcome [35]. In the Inter99 study, we have previously found that leptin, but not adiponectin or CRP, may play a mediating role in overweight-induced hypertension [36]. Therefore, adjustment for blood pressure could be considered an over-adjustment bias when relating leptin to CVD risk. However, as seen in Fig 2B, leptin did not decrease the BMI-associated CVD risk much, so again overall our study does not provide evidence that leptin plays an important role, in human CVD.

Of the adipose tissue-derived substances studied (directly or indirectly), the interleukin-6 product CRP was the only substance that was consistently significantly associated with outcome in all models. Although interleukin-6 can originate from many different tissues [8], it has been estimated that ≈30% of total circulating concentrations of interleukin-6 originates from adipose tissue in healthy persons [37]. Our data indirectly suggests that interleukin-6 originating from fat tissue may play a role in overweight- and obesity-related CVD, because CRP decreased the BMI-associated CVD risk markedly [38], and because it is well known that CRP itself is only a marker not a cause of CVD [39]. In the context of CRP and CVD, it is worthy of note that CRP predicted risk after adjustments for traditional and metabolic risk factor, including estimates of insulin resistance and HgA1c, and that CRP also predicted CVD in the non-smoking part of Inter99. Nevertheless, CRP did not change C-statistics, and although CRP did change NRI and IDI, the changes were small, less than 5% for NRI. Therefore, the usefulness of CRP as a risk marker in Inter99 was overall limited.

### Strengths and limitations

The main strengths of our study include its longitudinal population-based design, a relatively large number of randomly selected participants, and especially the availability of comprehensive data for multivariable adjustment for confounding factors. Thus, based on the PubMed database, only one other study was found which related adiponectin and leptin to incident CVD after adjustment for creatinine clearance [40]. Furthermore, the long-term follow-up, and the use of standardized registry-based diagnosis, which allows very high percentage of follow-up, are worth mentioning.

The limitations of the study include single measurements of biomarkers and the low initial participation rate of 52.5%, raising the possibility of recruitment bias. Toft et al. have extensively dealt with this topic, and they found that unhealthy lifestyle, bad health, and perceived susceptibility to disease among others were important mediators of participation in the Inter99 study [16]. We, therefore, suspect that the participants in Inter99 were unhealthier than non-participants, potentially biasing our results. We also have to admit that the results from our study population, aged 30 to 60 years, with a mean BMI of 26.3 ±4.6 kg/m2, may not be extrapolated to younger and older age groups with different prevalence of overweight and obesity. We also have to acknowledge that performing an observational cohort study within a randomized lifestyle intervention study, is a limitation, although we corrected for the intervention in our multivariable models. Nevertheless, this limitation appears to be minor, because the intervention in Inter99 did not translate into a lower risk of CVD [15]. In this context, it is worthy of note, that in model 2, baseline serum cholesterol (HR with 95%CI: 1.24 [1.20–1.39], P<0.001, per mmol/L increase), systolic blood pressure (HR with 95%CI: 1.15 [1.10–1.20], P<0.001 per 10 mmHg increase, and fasting plasma glucose, without diabetes mellitus in model 2 (HR with 95%CI: 1.10 [1.04–1.15], P<0.001, per mmol/L increase) were associated with CVD risk in Inter99, showing the risk predicting value of these traditional CVD risk factors in Inter99. Furthermore, it is worthy of note that we measured adipocytokine concentrations and not adipocytokine bioactivity, which may be relevant in a pathophysiological perspective. Finally, we feel the need to emphasize that this study was observational, and hence, causality cannot be established.

## Conclusion

Overall, the present study did not find evidence to suggest that adiponectin and leptin are useful biomarkers for increased CVD risk, and thus raises questions over their role in CVD. In contrast, the present study showed that CRP predict CVD risk independent of the Framingham Risk Score variables, and in a pathophysiological perspective, our results indicate indirectly that interleukin-6-related pathways may play a role in overweight- and obesity related CVD [41]^.^
